# The Autism Program Environment Rating Scale in Swedish Primary School: Cultural Adaptation and Content Validation

**DOI:** 10.1007/s10803-024-06544-7

**Published:** 2024-09-18

**Authors:** Klara Wenneborg, Lise Pettersson Roll, Sven Bölte, Samuel Odom, Hampus Bejnö

**Affiliations:** 1 Department of Special Education, Stockholm University, Stockholm, Sweden; 2 Center of Neurodevelopmental Disorders (KIND), Division of Neuropsychiatry, Department of Women’s and Childrens’ Health, Centre for Psychiatry Research, Karolinska Institutet & Region Stockholm, Stockholm, Sweden; 3 Child and Adolescent Psychiatry, Stockholm Health Care Services, Region Stockholm, Stockholm, Sweden; 4 Curtin Autism Research Group, Curtin School of Allied Health, Curtin University, Perth, Western Australia; 5 Frank Porter Graham Child Development Institute, University of North Carolina at Chapel Hill, Chapel Hill, USA

**Keywords:** Autism spectrum disorder, Content validity, Cultural adaptation, Learning environment, Primary school, Rating scale

## Abstract

There is a recognized need to improve inclusive learning environments for autistic children in primary school settings in Sweden. This study aimed to translate, cross-culturally adapt, and assess the content validity of the Swedish primary school version of the Autism Program Environment Scale (APERS), originally developed to evaluate autism program quality in educational settings in the United States. Following the translation into Swedish and the first cultural adaptation of the APERS, a content panel group of 14 professionals rated its content validity. Four of the content panel members also participated in individual interviews that provided a qualitative evaluation of the instrument’s content validity. Finally, the authors piloted the APERS in 10 Swedish primary school classrooms to assess its feasibility. The ratings and qualitative information from the content panel members indicated a substantial need for the Swedish APERS in primary school, resulting in the culturally adapted APERS-Primary-Sweden (SE). The instrument demonstrated a high level of cross-cultural content validity for assessing the quality of the learning environment for students with autism in Swedish primary school settings. The pilot testing of the instrument resulted in further cultural adaptations. In conclusion, APERS-Primary-SE is a comprehensive scale that can be used to assess the quality of primary school learning environments for children with autism in Sweden. Further research is needed to evaluate the adapted instrument’s effectiveness in improving the learning environment in Swedish primary school classrooms.

In recent years, there has been a growing emphasis on studying the benefits of environmental adaptations for individuals with autism^1^. This shift indicates a move towards a biopsychosocial perspective of disability and ability, diverging from the prevailing medical perspective that primarily focuses on individual deficits rather than considering the broader social and physical environment (Pellicano & den Houting, 2022). This change in perspective may be related to the substantial increase in autism diagnoses seen in many countries over the last decades, as indicated by various studies (Talantseva et al., 2023; Zeidan et al., 2022).

The current global prevalence of autism is reported to be about 1%, with the highest rates in the United States, followed by Sweden (Talantseva et al., 2023). A recent study in southwestern Sweden revealed a 1.1% autism diagnosis rate among 12-year-olds (Fast et al., 2023), while another report (Jablonska et al., 2022) found that 2.5% of children and youth in the Stockholm region had a registered autism diagnosis at some point during 2015–2020. The rising rate of autism diagnoses has not only increased awareness but has also highlighted needs beyond the traditional clinical settings, including workplaces and schools (Dreaver et al., 2020; Leifler et al., 2021).

Consequently, there has been a surge in research focusing on environmental barriers for autistic individuals in recent years (Leifler et al., 2021). This is exemplified in the work on determining the functional circumstances of autism using the World Health Organization International Classification of Functioning, Disability and Health (ICF; WHO, 2001). ICF is founded on a biopsychosocial model of functioning, exploring not only disabilities but also abilities, strengths, and environmental factors influencing individual functioning (Bölte, 2024). Research in this domain highlights the importance of interventions targeting environmental aspects, as exemplified in a scoping review by Krieger et al. (2018) that investigated supporting and hindering environments and drew attention to the environment as a crucial factor in participation.

Considering that the majority of children with autism attend mainstream schools in Sweden, it is essential for regular education to provide an inclusive learning environment, aligning with policy documents and conventions such as the Salamanca Statement (1994) and the United Nations Convention on the Rights of Persons with Disabilities (UN, 2008). However, despite explicit inclusion goals in international conventions and Swedish policy documents (Van Kessel et al., 2019), inclusive practices for autistic students remain limited in many mainstream schools (Pellicano et al., 2018).

In a study involving 4,778 school staff members across 68 schools from 13 municipalities in Sweden, Bölte and colleagues (2021) found that only 6% of the participants felt prepared by their professional education to teach students with neurodevelopmental conditions. Additionally, only 18% reported that specific supports were provided at a level aligning with students’ needs. In another Swedish study (Leifler et al., 2024), students with neurodevelopmental conditions, their parents, and teachers assessed their perceived level of educational inclusion. The results indicated even less favorable ratings among students and parents than among school staff. Teachers’ average ratings indicated a moderate level of inclusive practice, while the participating students and parents rated the level of inclusive practices as between moderate and doubtful. Furthermore, recent findings from youth cohort registers in Sweden show that only 57% of autistic students with average or above-average intellectual ability qualified for upper secondary school, in contrast with 86% of students not diagnosed with autism (Stark et al., 2021). Collectively, these findings highlight significant room for improvement in educational inclusion and overall educational quality for students with autism in Sweden.

Using observational instruments to assess variables contributing to educational quality is well-established in school research. However, efforts have predominantly focused on the quality of general early childhood education, with instruments such as The Inclusive Classroom Profile (Lundqvist & Larsdotter Bodin, 2021; Soukakou, 2016), Early Childhood Environmental Rating Scale 3rd ed. (Harms et al., 2015; Kärrby & Giota, 1994) and the Classroom Assessment Scoring System (Pianta et al., 2008). There are fewer assessment tools available to evaluate the quality of elementary school education, particularly when it comes to the learning environment for autistic students (Odom et al., 2022). In Sweden, there is currently no validated instrument designed to evaluate the quality of the learning environment for school-aged students with autism.

In the United States, Odom and colleagues (2018) developed the Autism Program Environment Rating Scale (APERS) to assess the quality of educational programs for autistic students. Program quality (i.e., the overall quality of the learning environment) includes factors such as the physical environment, learning climate, teaming, family involvement, and instructional approaches. The APERS has robust psychometric properties as an assessment tool (Odom et al., 2018), and use of the APERS has successfully shown solid effects in enhancing the quality of the learning environment for students with autism when applied as a part of an intervention in educational settings (Hume et al., 2022; Odom et al., 2013; Sam et al., 2021). The APERS is designed with the underlying rationale that educational program quality serves as the foundation for effective instruction and the use of evidence-based practices (Odom et al., 2013). The English version of the scale is available in two formats: Autism Program Environment Rating Scale Preschool/Elementary (APERS-PE; Odom et al., 2024) and Autism Program Environment Rating Scale Middle/High School (APERS-MH; Odom et al., 2023).

When using a rating scale in a country other than for which it was originally developed, it is important to translate and adapt it to align with the educational and cultural context of that country (Hilton & Skrutkowski, 2002; Sousa & Rojjanasrirat, 2011). During this cultural adaptation process, establishing content validity is an essential component. Content validity reflects how well the elements of the instrument represent the construct of interest, and it is usually established by relying on the knowledge of people who are familiar with the construct being measured (Almanasreh et al., 2022; Grant & Davis, 1997). For example, when assessing the quality of the learning environment for autistic students in Swedish school settings, it is crucial that the instrument captures this particular aspect and that those familiar with the context are consulted. Yet, few instruments used for diagnostic or intervention purposes in autism undergo content validity evaluation (Bejnö et al., 2019). Researchers often rely solely on face validity (Downing, 2006; Lynn, 1986) and disregard cross-cultural specificities.

A systematic process is essential to establish content validity (Rubio et al., 2003). In 2019, Bejnö and colleagues translated, adapted, and assessed the content validity of a Swedish version of the APERS-PE (Odom et al., 2018) for use in Swedish preschool settings where children with autism are enrolled. In the process and because of significant differences between Swedish preschool and elementary school (such as different curricula and different forms of organizations; Bejnö et al., 2019), the scale was adapted and designed specifically and only for preschool settings. It was named APERS For Preschool in Sweden (APERS-P-SE).

In Sweden, preschool is voluntary and intended for children aged 1 to 5 years; compulsory schooling begins at age six, and the educational settings of the two differ substantially. As previously mentioned, there is currently no age-appropriate, scientifically and culturally sound rating scale available in Sweden to assess the learning environment for autistic children enrolled in any of the stages in the Swedish compulsory schooling system. The aim of the current study was, therefore, to translate, adapt, and evaluate the content validity of the APERS-PE (Odom et al., 2018, revised 2020) for use in Swedish primary schools (preschool class [i.e., the first year of school in Sweden] to third grade).

## Method

### Design and Procedure

**Fig. 1:**
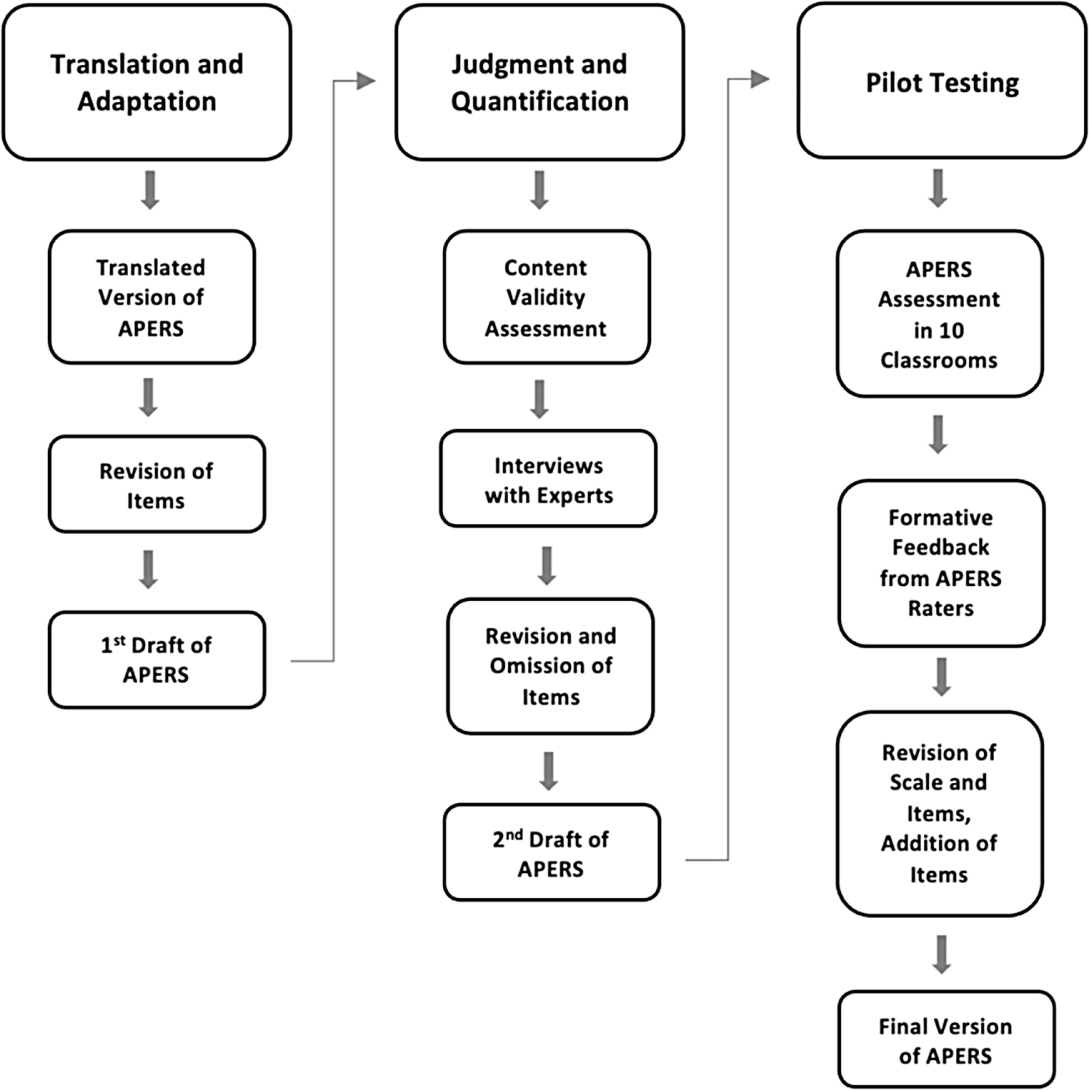
Adaptation process in the development of the Swedish primary school version of APERS

The current study was ethically approved by the Swedish Ethical Review Authority (2021–04315) and pre-registered on Open Science Framework (10.17605/OSF.IO/NWGTB) as a part of a larger research project (ClinicalTrials.gov; #NCT05509309). A mixed methods approach (Wisdom & Creswell, 2013; Johnson et al., 2007) was employed in the study to guide the adaptation and content validation of the Swedish primary school version of APERS. Adaptation and content validation were addressed in three phases: (1) translation and adaptation, (2) judgment and quantification, and (3) pilot testing. Amendments were made after each phase in a stepwise procedure, as illustrated in Fig. 1. Ratings from content panel members were collected in a content validation process (Almanasreh et al., 2019). Both the ratings and qualitative information from each phase influenced the subsequent phases, resulting in the culturally adapted Swedish version of the APERS-PE, the APERS-Primary-Sweden (SE). The procedure is described step by step under each respective heading.

### Instrument

The APERS is an assessment tool designed to evaluate the educational quality for autistic students. It draws on observations, interviews, and reviews of student documentation to generate ratings of 10 key domains, contributing to an overall assessment of the learning environment. As previously noted, the original English version of APERS is available in two formats: APERS-PE for preschool and elementary students and APERS-MH for middle-school and high-school students. In 2019, APERS-PE was translated and adapted specifically for the Swedish preschool context, resulting in the APERS-P-SE (Bejnö et al., 2019). In the current study, the latest version of APERS-PE, revised in 2020 and consisting of 62 items, served as the foundation for the translation and adaptation process, with the aim of creating a Swedish version tailored for use in primary classrooms.

When assessing the quality of the learning environment, APERS-PE items are rated on a 1 to 5-point Likert scale, with a score of “1” indicating poor quality and “5” indicating high quality. The items are grouped into 10 domains (e.g., Positive Learning Climate) and 37 subdomains (e.g., Staff-Student Interactions); see Table 2 for all domains and subdomains. The APERS yields a total mean item-rating score, conceptualized as overall autism program quality, as well as separate scores for domains, subdomains, and items. Professionals working in schools (e.g., special education teachers or other professionals within the student health services) can be trained by experienced APERS raters to use the instrument. To conduct an APERS assessment, data is collected through structured observations in classrooms and other school settings where students with autism are engaged.

Furthermore, to complement observations, interviews are conducted with school staff (e.g., teachers, teacher assistants, and special educators) and parents or caretakers of autistic students. Finally, documents such as Individual Education Plans containing individualized goals and accommodations for target students are reviewed. The combined information from the observation, interviews, and documentation review is then used as the foundation for rating each APERS item.

### Translation and Adaptation

The revised version of APERS-PE (2020) was translated from English to Swedish by a Swedish PhD-level clinical psychologist familiar with the APERS, fluent in English and Swedish, with in-depth knowledge of autism and proficiency in scale development and psychometrics. The translation was done with approval from the original authors (the National Professional Development Center on Autism Spectrum Disorder [NPDC]; Odom et al., 2013).

#### Development of First Draft

Following the translation, the authors of the current article discussed and provided internal feedback regarding the appropriateness of the translation and the contents of each item in close collaboration with the original APERS authors. During this collaborative step, the research group compared the translation with the original version. This resulted in some modifications, such as changes in wording and precise content. For example, the terms “grades” and “autism team” were omitted because Swedish schools do not give grades until sixth grade and do not have specific autism teams. Thus, the authors of the current article agreed on adaptations to produce a first draft of the Swedish primary school APERS, the APERS-Primary-SE.

### Judgment and Quantification

The judgment and quantification stage involves inviting a panel of experienced professionals or stakeholders with in-depth knowledge of the content area to assess instrument elements (Almanasreh et al., 2022). The most common approach to quantify an instrument’s content validity is the Content Validity Index (CVI). The CVI is based on the content panel members’ ratings for each item, assessing their relevance and representativeness to the content area, typically on a 4-point Likert scale from “0” (e.g., no relevance/representativeness) to “3” (e.g., full relevance/representativeness; Almanasreh et al., 2022; Rubio et al., 2003). When assessing content validity, it is important to have a mix of perspectives in the content panel and select those who are not hesitant to give feedback and offer criticism (Slocumb & Cole, 1991). Recommendations on the number of content panel members vary, ranging from 3 to 20 (Lynn, 1986; Polit et al., 2007; Rubio et al., 2003). The final decision on the number of content panel members needed depends on the desired expertise and the representation range of the panel (Grant & Davis, 1997).

#### Participants – Content Panel Members

To assess the content validity of the Swedish APERS for primary school, 14 professionals (nine female, five male) with in-depth knowledge of autism and the Swedish school context were recruited, two of whom also were parents to a child with autism (see Table 1). Our inclusion criteria were: (1) being very well-oriented in the content area (as informally assessed by the authors), (2) being a professional within the Swedish school context, and/or (3) being a stakeholder in the same area. None of the content panel members had previous experience of using APERS. The panel members were offered a compensation of ~$150 (SEK 1500) to participate due to the comprehensiveness of the rating and reviewing process. Four of the panel members (three female and one male) were also interviewed after they had completed the content validity rating. The four interviewees were selected to represent the whole group as comprehensively as possible; they were from different employment settings, had more than 15 years of professional experience within the Swedish education context, and one was also a parent of an autistic child.

**Table 1:** Participant characteristics for the content panel members (*n* = 14)

Participant characteristic	*n*	%
Gender		
Female	9	64
Male	5	36
Employment*		
School	3	21
Privat practice	5	36
Habilitation center	2	14
University	2	14
Other**	4	29
Years of experience in content area		
5–10 years	3	21
15–20 years	6	43
20 + years	5	36
Education/degree*		
Special Educator	4	29
Master’s degree in Special Education	4	29
Licensed Psychologist	6	43
Specialist in Educational Psychology	2	14
Licensed Speech and Language Pathologist	2	14
PhD student within content area	2	14
Board Certified Behavior Analyst (BCBA)	2	14
Other	1	7

*Some participants had more than one employment/education. Therefore, the totals in those sections are higher than the sample size, and the percentages sum to greater than 100%

**E.g., central position at municipality or position at National Agency for Special Needs Education

#### Procedure – Content Validity Assessment

Before rating the scale’s content validity, panel members received a cover letter with a written summary outlining the study’s aims, reviewer instructions, and a content review questionnaire. Additionally, all members of the content panel were contacted via phone or digital meeting to obtain the information verbally and to have the opportunity to ask clarifying questions. Subsequently, all members of the content panel received a copy of the first draft of the Swedish APERS translated and adapted from the APERS-PE 2020. The panel members rated each item’s level of clarity and comprehensiveness as well as each subdomain’s relevance to its superordinate domain (e.g., Staff-Student Interactions to Positive Learning Climate) and the relevance of each domain (e.g., Positive Learning Climate) to the scale in its entirety (Grant & Davies, 1997). Item level clarity and comprehensiveness were rated on a 4-point Likert-scale (0 to 3) ranging from “not clear/comprehensive (0), “major revisions needed” (1), “minor revisions needed” (2), to “clear/comprehensive” (3). Relevance was also rated on a 4-point Likert-scale (0–3) ranging from “not relevant” (0), “not relevant without revision” (1), “relevant but needs minor revision” (2) and “very relevant and exhaustive” (3). Panel members were also given the opportunity to provide written feedback on item, subdomain, domain, and whole-scale levels to elaborate on their ratings.

Content panel members additionally rated six general statements (for all statements, see Table 4) regarding their perception of APERS in its entirety, the scale’s usefulness, relevance, need, necessity, and practical use. The statements were rated on a scale from 0, “I do not agree”, to 3, “I completely agree”.

#### Procedure – Interviews

In addition to completing the content review questionnaire, four of the content panel members were also interviewed about APERS content validity. This was done to investigate their responses further and to collect a more in-depth source of information. Two overarching questions guided the interviews: (1) What are the content panel members’ general impressions of the APERS? and (2) How can the APERS be improved to enhance the scale’s relevance and usefulness in Swedish primary school settings? The first author (K.W.) conducted the interviews using a semi-structured interview guide (available as Supplementary Material 1 online) developed by the research team. The respondents were asked to elaborate on specific written comments they had provided. Follow-up questions were posed when needed. All interviews were recorded via digital audio recording and transcribed verbatim afterward. The duration of the interviews varied between 36 and 43 min.

#### Second Draft

All members of the research group reviewed the results of the ratings, the written comments, and the transcribed interviews. Based on this, further adaptations were made, including revision of wordings and terminology in the adapted scale based on the content panel members’ comments. Furthermore, three items in the domain Teaming were removed, as many panel members commented that team structures look different in the Swedish school compared to the United States. The research group then agreed on a second Swedish draft of the APERS-Primary-SE. All adaptations in this and the subsequent steps were done in agreement with the original authors of the APERS, including recurring meetings, discussions, and minor revisions on specific wordings based on suggestions by the original authors (NPDC; Odom et al., 2013).

### Pilot Testing

Following the finalization of the second draft of the APERS-Primary-SE, the scale was piloted for feasibility in 10 different classrooms in a Swedish primary school. To prepare for piloting, three special educators employed at the target school and one affiliated special educator participated in a four-day training to become coaches within the pilot study. The training, adapted from the National Professional Development Center on Autism Training Model (Waligórska et al., 2019), covered learning environment quality for learners with autism, assessment of the learning environment in classrooms using the APERS, selecting and designing goals based on the assessment, choosing and using evidence-practices to meet those goals, and the development of an action plan to improve the quality of the learning environment. Subsequently, the coaches conducted baseline assessments with the APERS-Primary-SE in collaboration with the current study’s first (K.W.) and last (H.B.) author (i.e., conducting most assessments in pairs, including one coach and one researcher). The assessments were then used to guide and support improvements in the learning environment. The coaches assessed between two and four classrooms each, both at the beginning (pre-assessment) and at the end (post-assessment) of one school semester. Drawing from their experience using the APERS-Primary-SE, the coaches provided feedback about content validity. They highlighted specific items and domains they believed needed to be further modified to make the instrument fully applicable, useable, and relevant in applied school settings.

#### Final Version

**Fig. 2:**
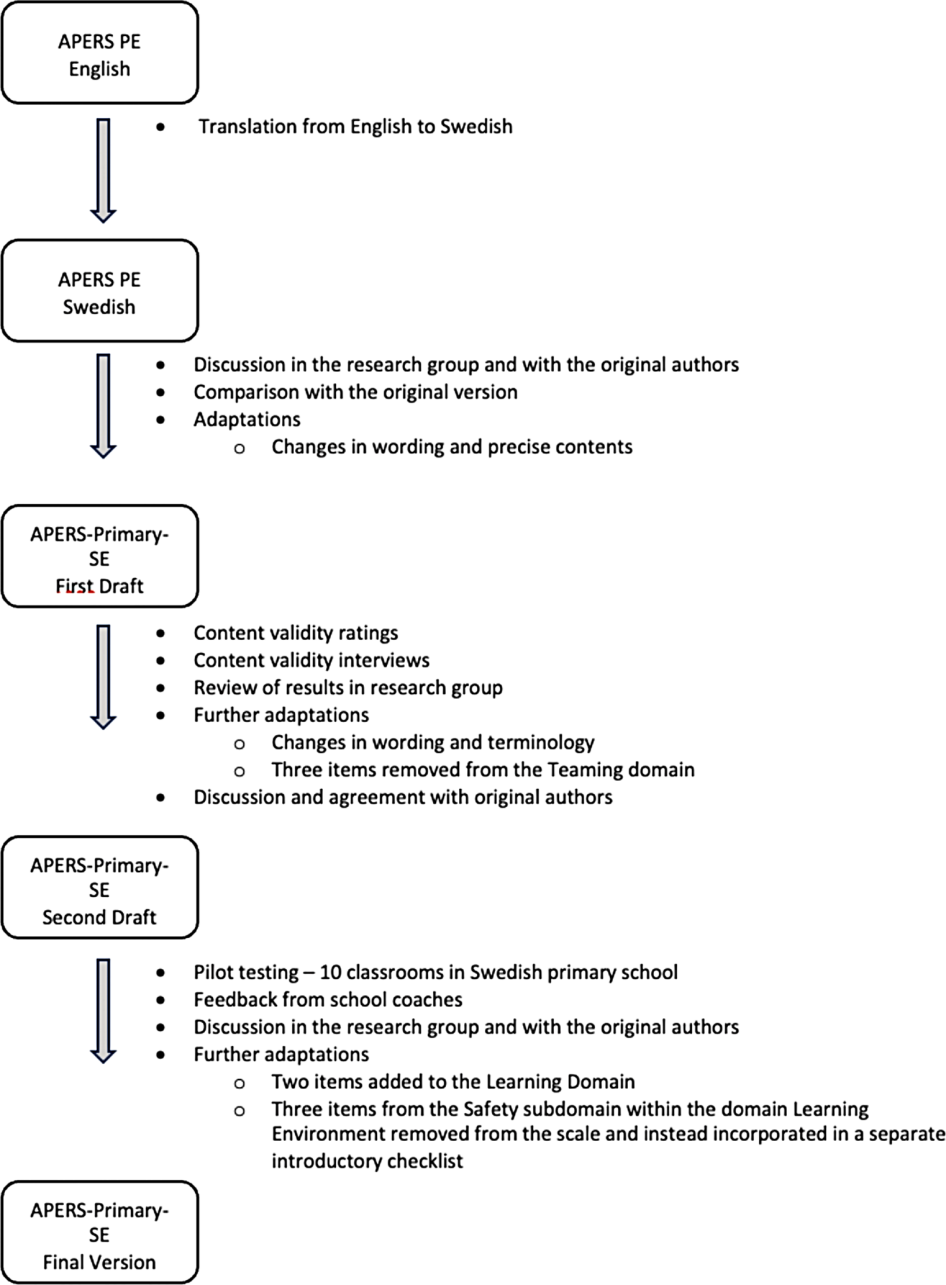
Overview of the revisions made in each phase during the development of the Swedish primary school version of APERS

After the pilot study was completed, all feedback from the coaches was compiled and internally discussed within the research team. Suggestions for necessary adaptations were presented and discussed with the original authors, and revisions were made accordingly. For an overview of the revisions in each phase of the adaptation, see Fig. 2.

### Analyses

#### Content Validity Index

To quantify the content validity ratings completed by the 14 content panel members, the Content Validity Index (CVI; Rubio et al., 2003) was employed. CVI is usually calculated based on content panel members’ ratings of an instrument’s relevance, representativeness, comprehensiveness, and/or clarity relative to the targeted measurement construct (Grant & Davis, 1997; Lynn, 1986; Rubio et al., 2003). In the current study, CVI was calculated in concordance with established recommendations (Rubio et al., 2003) based on the 4-point Likert scale scores described under the section Judgement and Quantification. A CVI was first calculated for all APERS-Primary-SE items by counting the number of panel members who rated the items’ level of clarity and comprehensiveness as “2” or “3” and then dividing it by the total number of panel members (i.e., providing the proportion of panel members evaluating each item as clear and comprehensive).

CVIs were also calculated for all subdomains’ relevance for their superordinate domains (See Table 2) by averaging mean CVIs across all subdomains. Finally, CVIs were calculated for all domains’ relevance for the whole scale by averaging mean CVIs across all domains. The CVI classification defined by Lynn (1986) was used to interpret the findings on item, subdomain, and domain level, where a CVI threshold of ≥ 0.78 is deemed a sufficient level of content validity when there are at least nine panel member raters.

**Table 2:** Mean CVI (max. 1) and mean values (max. 3) of content panel members’ ratings on relevance for subdomains to their domain

Domain	Subdomain	Items	CVI subdomains relevance	Mean rating subdomains relevance	CVI item clarity	Mean rating item clarity	CVI item comprehensiveness	Mean rating item comprehensiveness
Total scores		1–62	0.99	2.86	0.96	2.77	0.97	2.80
Learning Environments		1–9	0.99	2.83	0.96	2.71	0.97	2.72
	Safety	1–3	0.93	2.50	0.93	2.76	0.98	2.74
	Organization of Learning Environments	4–5	1.00	3.00	1.00	2.57	1.00	2.71
	Materials	6–7	1.00	2.86	0.93	2.68	0.96	2.71
	Visual schedules	8	1.00	2.93	1.00	2.86	0.86	2.71
	Transitions within the school day	9	1.00	2.86	1.00	2.79	1.00	2.79
Positive Learning Climate		10–14	0.93	2.79	0.99	2.79	1.00	2.80
	Staff-Student Interactions	10–11	1.00	3.00	1.00	2.79	1.00	2.79
	Staff behaviors	12–13	1.00	2.93	0.96	2.75	1.00	2.75
	Promoting Diversity	14	0.79	2.43	1.00	2.86	1.00	2.93
Assessment & IEP Development		15–20	0.98	2.80	0.90	2.60	0.92	2.70
	Assessing Student Progress	15	1.00	3.00	0.86	2.43	0.79	2.50
	Assessment Process	16	1.00	2.79	0.86	2.71	0.93	2.86
	IEP Goals	17–19	0.93	2.64	0.90	2.60	0.93	2.69
	Program Transition Planning	20	1.00	2.79	1.00	2.64	1.00	2.79
Curriculum & Instruction		21–32	1.00	2.95	0.99	2.83	0.99	2.86
	Instructional Format	21, 26	1.00	3.00	1.00	2.75	1.00	2.71
	Instructional Content	22, 25	1.00	2.93	1.00	2.86	1.00	2.89
	Instructional Strategies	23–24, 27–32	1.00	2.93	0.98	2.86	0.97	2.88
Communication		33–37	1.00	2.95	0.99	2.86	0.99	2.83
	Planning for Communication	33	1.00	2.86	1.00	2.79	1.00	2.86
	Communication Rich Environment	34	1.00	3.00	1.00	2.93	1.00	2.86
	Individualized Communication Instruction	35	1.00	3.00	1.00	2.79	1.00	2.93
	Responsiveness to Student Communication	36	1.00	3.00	0.93	2.86	0.93	2.71
	Communication Systems	37	1.00	3.00	1.00	2.93	1.00	2.79
Social Competence		38–42	0.98	2.79	0.96	2.81	0.96	2.77
	Arranging Opportunities	38–39	1.00	2.86	0.96	2.82	0.96	2.75
	Teaching and Modeling	40	1.00	2.86	1.00	2.71	1.00	2.86
	Social Skills Instruction	41	1.00	2.86	0.93	2.79	0.93	2.79
	Peer Social Networks	42	0.93	2.57	0.93	2.93	0.93	2.71
Personal Independence & Competence		43–46	1.00	2.96	0.98	2.79	0.96	2.88
	Personal Independence	43–45	1.00	2.93	0.98	2.81	0.95	2.86
	Self-Management	46	1.00	3.00	1.00	2.71	1.00	2.93
Interfering behavior(s)		47–51	1.00	2.89	0.97	2.84	0.99	2.91
	Proactive Strategies	47	1.00	3.00	1.00	2.79	1.00	2.86
	Behavioral Assessment	48–49	1.00	2.79	0.96	2.86	0.96	2.93
	Behavior Management	50	1.00	2.86	0.93	2.79	1.00	2.93
	Data Collection	51	1.00	2.93	1.00	2.93	1.00	2.93
Family Involvement		52–55	0.98	2.86	0.96	2.89	0.98	2.95
	Teaming	52	1.00	2.93	0.93	2.79	1.00	3.00
	Communication	53–54	0.93	2.79	0.96	2.89	0.96	2.93
	Parent-Teacher Meetings	55	1.00	2.86	1.00	3.00	1.00	2.93
Teaming		56–62	1.00	2.86	0.91	2.67	0.94	2.72
	Team Training	56	1.00	2.71	0.93	2.50	0.93	2.57
	Team Membership	57–59	1.00	2.86	0.90	2.69	0.90	2.67
	Team Meetings	60–61	1.00	2.86	0.93	2.82	0.93	2.93
	Implementation	62	1.00	3.00	0.86	2.50	0.93	2.64

In addition to computing CVIs for items, subdomains, and domains, mean ratings were calculated for comprehensiveness and clarity on item level and for relevance on subdomain and domain levels. Lastly, mean values for the panel members’ ratings of the scale in its entirety (e.g., “I believe that APERS-Primary-SE is a relevant scale to assess the learning environment for children with autism in Swedish school”) were calculated by dividing the total score of each of the six statements by the total number of panel members (*n* = 14).

#### Thematic Analysis

The written comments from the 14 content panel raters and the verbal data from the four transcribed interviews were analyzed using thematic analysis (Braun & Clarke, 2019). All data was coded and processed using NVivo 13 (Lumivero, 2020). An inductive approach was applied to address two overarching questions: “What are the content panel members’ general impressions of the APERS?” and “How can the APERS be improved to enhance the scale’s relevance and usefulness in Swedish primary school settings?”. Segments of texts were assigned codes after in-depth readings of the written comments and transcribed verbal data, leading to the generation of concepts and themes (Azungah, 2018).

To increase the trustworthiness of the identified themes, both the first author (K.W.) and the last author (H.B.) independently followed the systematic and stepwise approach outlined by Braun and Clarke (2006, 2013). All transcribed interviews and written comments were read multiple times to generate initial thoughts and ideas. Codes were then assigned for text units of interest, followed by analysis of all coded text segments, resulting in the identification of initial themes. The themes were matched with the coded text segments and the complete set of original interviews and comments, and general themes were subsequently identified and named. After that, the two independent coders jointly re-evaluated themes for all written comments and interviews, reaching a consensus on concluding themes and names. Lastly, the rest of the research group agreed on the conclusion and analysis drawn from the data.

## Results

### Content Validity Index

Table 2 lists the CVI mean ratings of items’ clarity and comprehensiveness. They varied between 1 and 3 (on a scale of 0 to 3). All single items received a CVI above the validity threshold of 0.78 for both clarity and comprehensiveness.

#### Item Scores for Clarity and Comprehensiveness

The mean ratings for item clarity (as shown in Table 2) were 2.77, ranging from 2.43 to 3.00. The mean ratings for item comprehensiveness were 2.80, ranging from 2.50 to 3.00. When examining content panel members ratings at the domain level, the Assessment and IEP Development domain received the lowest ratings for both clarity (*M* = 2.60) and comprehensiveness (*M* = 2.70), while the domain Family Involvement received the highest ratings (clarity *M* = 2.89 and comprehensiveness *M* = 2.95).

#### Subdomain Scores for Relevance

The mean rating for all subdomains for their relevance to their respective domains (see Table 2) was 2.86, with a range of 2.57–3.00. The subdomain Peer Social Networks received the lowest ratings for relevance to its domain (Social Competence, *M* = 2.57), while numerous subdomains, particularly those under the Communication domain, were conclusively judged “very relevant” (*M* = 3.00) to their overarching domain by all content panel members.

#### Domain Scores for Relevance

The mean rating of all domains’ relevance for the overall learning environment quality in Swedish school was 2.88 (range 2.64–3.00); see Table 3. Family Involvement, Learning Environments, and Assessment & IEP Development produced the least high scores (Family Involvement *M* = 2.64, the other two *M* = 2.79). Three domains, Curriculum & Instruction, Communication, and Social Competence, were rated with the highest level of relevance across all raters (*M* = 3.00).

**Table 3:** CVI values (max. 1) and Mean ratings of domains’ relevance (max. 3) for the whole scale

Domain	CVI	Relevance
Learning environments	1.00	2.79
Positive learning climate	1.00	2.93
Assessment & IEP Development	0.93	2.79
Curriculum & Instruction	1.00	3.00
Communication	1.00	3.00
Social Competence	1.00	3.00
Personal Independence & Competence	1.00	2.93
Interfering behavior(s)	1.00	2.86
Family Involvement	1.00	2.64
Teaming	1.00	2.86
Total score	0.99	2.88

### Overall Ratings of APERS-Primary-SE

The mean of the six ratings about the APERS-Primary-SE in its entirety (personal preference, usefulness, relevance, need, necessity, and practical use) was 2.60 (range 1.86–3.00; see Table 4). The ratings regarding the need for a scale such as APERS-Primary-SE and the previous lack of a scale to assess the learning environment for autistic students in the Swedish school yielded the highest scores (*M* = 3.00 and *M* = 2.86). The rating that yielded the lowest score (*M* = 1.86) concerned whether it was realistic to practically use APERS-Primary-SE in the Swedish school to rate the learning environment.

**Table 4:** Mean values (max. 3) and Standard Deviations of content panel members’ ratings on whole scale statements

Statement	Content panel members’ rating
1. I believe that APERS-Primary-SE is a relevant scale to assess the learning environment for children with autism in Swedish school	2.79 (0.41)
2. I believe that APERS-Primary-SE is a useful scale to assess the learning environment for children with autism in Swedish school	2.43 (0.73)
3. I believe that there is a need for a rating scale such as APERS-Primary-SE to assess the learning environment for children with autism in Swedish school	3.0 (0.00)
4. I believe that it is realistic to practically use APERS-Primary-SE to assess the learning environment for children with autism in Swedish school	1.86 (0.74)
5. I regard APERS-Primary-SE as a scale that I would like to use to assess the learning environment for children with autism in Swedish school	2.64 (0.61)
6. I believe that there has been a lack of a good rating scale to assess the learning environment for children with autism in the Swedish school	2.86 (0.52)

### Thematic Analysis of Interviews and Written Comments

To answer the two overarching questions guiding our interviews and the analysis of the written comments, three themes were generated: *Terminology*,* Comprehensiveness*, and *The need for APERS*. The three themes will be summarized in the following section, with quotations to give voice to the content panel members’ opinions.

#### Terminology

A recurring theme among the content panel members was the importance of using terminology applicable to the Swedish school system. The panel members stressed the importance of clarity, pointing out words and terms being unclear or unknown and terms that needed to be operationalized (e.g., “Team meetings – unsure if it is used and what is meant by this.”). The panel members also addressed the need to match terminology with governing documents and guidelines, pointing out differences between the United States and the Swedish school system. One participant wrote: “It is relevant, but certain parts would need to be more clearly linked/aligned, etc., with the governing documents, because otherwise, people may not use it, or may not want to use it – there might be resistance.”

#### Comprehensiveness

Most content panel members’ general impression of the APERS centered around the comprehensiveness of the instrument. While some panel members regarded this as a strength, others were concerned that it is too comprehensive regarding the lack of time and resources in schools. One participant stated: “Comprehensive is the word that comes to mind. And when you are aware of the time constraints in schools, that is what makes you feel hesitant about to what extent they will use it.” Many of the panel members suggested a desire for a shortened version of APERS since they recognized the need for the instrument but were concerned that it might not be used due to its length and complexity. One of the panel members wrote: “I think it would be difficult to conduct such a comprehensive observation. A version with fewer items would be easier. At the same time, everything is relevant…”.

#### The Need for APERS

Many of the panel members stressed the need for APERS in the Swedish school context and that there is a lack of a similar instrument in Sweden. In particular, several panel members reiterated that the APERS places demands and sets expectations on the school that are much needed for autistic students. One participant wrote: “It covers multiple areas. Good to have different areas/progression in the items. Can be an *eye-opener* for staff.” and another: “Particularly important to highlight everything that doesn’t happen during class time, highlighted well here. And the connection to the family is clear and significant.”

### Formative Feedback

Based on feedback from the four coaches participating in the pilot testing, two items related to individual adaptation of materials and seating arrangement were added to the domain Learning Environment. Both items were inspired by contents in an assessment tool for accessible education developed by the Swedish National Agency for Special Needs Education (Specialpedagogiska skolmyndigheten, 2018). Furthermore, based on feedback from coaches, three items from the Safety subdomain within the domain Learning Environment were removed from the scale. Instead, they were incorporated into the final version of the APERS-Primary-SE as a separate introductory checklist. This decision was made due to consistently high ratings across all safety items, which skewed the overall domain score and misrepresented the actual learning environment. This resulted in the final version of the Swedish primary school scale: APERS-Primary-SE.

## Discussion

In Sweden, the majority of children with autism attend mainstream schools, where previous research indicates limited inclusive practices (Bölte et al., 2021; Leifler et al., 2024; Stark et al., 2021), highlighting the need for quality reviews. Valid, cross-culturally adapted, and psychometrically evaluated rating scales designed to assess the quality of the learning environment for school-aged autistic students could potentially support inclusive practices of high quality for these students. However, such a scale is currently not available in Sweden. Although the original version of the APERS encompasses both preschool and elementary school, the significant differences between Swedish preschools and elementary schools necessitated the development of distinct versions for preschool (Bejnö et al., 2019) and primary school. The current study, therefore, focused on developing a version suitable for Swedish primary school classrooms by translating, cross-culturally adapting, and evaluating the content validity of a Swedish version of the APERS-PE (Odom et al., 2018, revised 2020), designed to assess learning environment quality in Swedish primary school settings for students with autism.

### Findings

Our results confirmed the content validity of the APERS-Primary-SE, affirming its relevance for use in Swedish primary school classrooms. Additionally, the results emphasize a significant need for a scale like APERS in Sweden.

There are several similarities between the results of this study and the previous study by Bejnö and colleagues (2019). In both adaptation processes, substantial modifications were needed to align the original APERS with the Swedish preschool and primary school contexts. For instance, in Sweden, there are no specific autism teams in either preschools or primary schools, and many professions included in the original APERS, such as speech and language pathologists and occupational therapists, are usually not part of either the Swedish preschool or school system. If present at all, these professionals mainly act as external supervisors, providing support on a sporadic basis.

Other significant adaptations were made to align the wordings of the scale with the terminology found in Swedish governing documents and guidelines, ensuring that the language used in the scale matches that typically employed in preschools and schools. This emphasis on alignment was highlighted as significant by the members of the content panel, as they pointed out that terminology and words not commonly used in schools could elicit resistance towards the instrument and create uncertainty among users.

Another finding in the current study that aligns with the findings of Bejnö and colleagues (2019) pertains to the feedback from content panel members suggesting that the instrument might be too comprehensive, considering time and resource constraints in schools. However, it should be noted that none of the participating content panel members in either of the studies had actually tested the scale in a preschool or school environment; they had only reviewed it. There might be a difference in attitudes when using the scale in a real school setting, as indicated in a qualitative study by Bejnö and colleagues (2023). In this study, stakeholders who participated in research aimed at improving the preschool learning environment using the APERS-P-SE were interviewed about their experiences. The findings from the qualitative study suggest that the APERS-P-SE-based model was perceived as significantly improving the quality of the learning environment in the participating preschools. Moreover, the study suggests that implementing an APERS-based model in the Swedish preschool support system is feasible and beneficial (Bejnö et al., 2022).

### Strengths & Limitations

Generally, thorough cultural adaptations of instruments are rare, and translation alone is typically the standard (Sousa & Rojjanasrirat, 2011). A strength of the present study is that it addressed both content validity and cultural adaptation in a comprehensive three-step process, with improvements made after each phase. To ensure a diverse representation, the content validity assessment involved the participation of 14 content panel members from various professions and professional settings. In addition to the process used when APERS was adapted to the Swedish preschool context (Bejnö et al., 2019), the judgment and quantification phase included not only ratings and written comments but also interviews conducted with a subset of the panel members. This allowed for both quantitative and qualitative analyses, as suggested in content validity literature (Almanasreh et al., 2019).

This mixed methods approach was used to better understand the connections or possible inconsistencies between qualitative and quantitative data and to allow participants to share their experiences across the research process (Wisdom & Creswell, 2013). To improve the scale’s applicability, usefulness, and relevance, a third and last step of pilot testing was introduced in the adaptation process. This step led to further adjustments, and a lesson learned is that while content validity ratings were generally high, relying solely on the translation and initial adaptations by the research group was not sufficient in itself. Therefore, it is important not only to consider overall high content validity ratings but also to put the instrument to practical use to produce an adapted scale with maximized relevance and applicableness.

This study has several limitations. Due to the comprehensive nature of the instrument, the Swedish version of APERS was not back-translated, with the permission of its original authors. Although the first, second, and last authors, all proficient in both Swedish and English and trained to administer APERS-PE, thoroughly reviewed the Swedish translation, a back translation might have improved the accuracy of the language translation (Gjersing et al., 2010). On the other hand, more than a single back translation is needed to ensure a valid and reliable instrument in the target language, as it may overlook nuances of meaning and cultural biases. Some researchers instead suggest a collaborative approach, assembling a team familiar with the cultures involved in the research and proficient in the respective languages (Douglas & Craig, 2007). In our case, we applied such a collaborative method, working closely with the original authors from the United States to ensure the instrument’s validity. Furthermore, it is advisable to conduct pre-testing with actual users to identify any necessary modifications before finalizing the instrument (Acquadro et al., 2008; Douglas & Craig, 2007). During our pilot testing phase, we implemented this step and made final adjustments in consultation with the original authors.

Apart from content validity, this study did not assess other psychometric properties of the Swedish APERS. Future research directions could include additional psychometric studies, such as reliability (test-retest) and various other forms of validity (e.g., construct, prognostic, criterion-related). However, to make meaningful examinations of those properties, it is crucial to first establish a content-validated cultural adaptation. Another limitation concerns the amount of community involvement. The current study was not fully participatory (Fletcher-Watson et al., 2019) but rather research- and education-driven, although parents of children with autism participated in the content panel group. In future studies aimed at adapting the APERS for older students, it would be beneficial to increase community involvement by inviting autistic students to participate in the research group.

While not inherently a limitation, APERS-Primary-SE is comprehensive and resource-intensive, as also highlighted by the content panel members. Evaluating a program’s quality, or all aspects of the learning environment, requires observing most or all scheduled activities throughout the school day. It also involves collecting data from various sources, which are essential for rating each APERS-Primary-SE item. This broad assessment is vital to derive meaningful information that is likely to influence overall learning environment quality for autistic students. A shorter and simplified version of the APERS-Primary-SE, as suggested by some of the content panel members, might be easier to implement but could compromise the instrument’s validity and usefulness.

### Future Directions

To address the concerns that the full instrument may not be used as intended or at all due to the significant time required, future research could investigate the utility of employing APERS-Primary-SE at the domain level to focus on specific areas for evaluation and intervention. On the other hand, it might not be the comprehensiveness of APERS-Primary-SE that poses the problem but rather the lack of time and resources in Swedish primary schools. The concerns raised by panel members were not mainly about the scale itself but were more related to time constraints and lack of personnel. Therefore, further research should involve implementing the APERS-Primary-SE in real school settings to evaluate its practicality and effectiveness within these time and resource constraints. Such an effort could provide valuable insights into the scale’s feasibility for school use.

To further explore the usefulness of APERS-Primary-SE, future research could use APERS assessments to improve quality and monitor potential changes in the learning environment through a between-group design study. Additionally, it would be valuable to evaluate the suitability of APERS-Primary-SE in fourth to sixth-grade settings, where the curriculum aligns more closely with primary school than preschool. Given the complexity of implementing new practices and interventions in schools, it would be appropriate for such studies to include both quantitative and qualitative data. This approach enables the evaluation of complex aspects, such as stakeholders’ perception of the intervention, which is crucial in outcome research (Curry et al., 2009).

Another area for future research is examining the utility and appropriateness of APERS-Primary-SE in other national contexts within Scandinavia. While the adaptation is tailored to the Swedish context, it is worth noting that Scandinavian countries share many similarities in their educational systems compared to the difference between Scandinavian countries and the United States. Although the adaptation may be particularly relevant for countries like Denmark and Norway (where the Swedish preschool version of the APERS has already been used in its current Swedish translation and adaptation), its generalizability to other national contexts remains to be determined and warrants further research.

## Conclusions

In summary, the APERS-Primary-SE is a comprehensive rating scale demonstrating a high level of content validity for assessing the quality of the learning environment for autistic students in primary school settings in Sweden. More research is needed to better understand the scale’s value and usefulness in real school settings. Other areas for future research could involve investigating generalizability to other Scandinavian countries, as well as whether a simplified version of APERS could prove useful without compromising the scale’s validity. However, as pointed out by participating content panel members, one cannot simplify an assessment to the extent that changes needed to improve the quality of the learning environment remain unnoticed. Thus, making a meaningful difference for students with autism in the Swedish school may require structural changes within the school system, and a comprehensive assessment instrument such as APERS-Primary-SE could play an important role in that process.

## Footnotes

1 We will use both person-first and identity-first language in this article, in accordance with different preferences in the autism and research communities (Buijsman, 2023).
